# Permanganate of Potassium in the Treatment of Vulvo-Vaginitis in Little Girls, and Gonorrhœal Metro-Vaginitis in Women

**Published:** 1894-02

**Authors:** 


PERMANGANATE OF POTASSIUM IN THE TREATMENT OF VULVO-VAGINITIS IN LITTLE GIRLS, AND GONORRHEAL METRO-VAGINITIS IN WOMEN.—In children the injection is made with the child in the gynecological position. A soft rubber catheter is introduced high up into the vagina, using a 1-4000 solution at first and gradually increasing the strength to 4-1000. A fountain syringe should be employed, and about a pint of the solution used at each injection, which should be repeated every other day. At first the discharge may be increased under the treatment, but the disease is usually cured permanently in about three weeks.

For gonorrheal metro-vaginitis in women a 1-1000 solution of permanganate of potassium is injected into the vagina twice daily. When the cervical and uterine canals are sufficiently patent they should be swabbed out with cotton saturated with the same solution.—*Ex.*

